# High Local Diversity of *Trypanosoma* in a Common Bat Species, and Implications for the Biogeography and Taxonomy of the *T. cruzi* Clade

**DOI:** 10.1371/journal.pone.0108603

**Published:** 2014-09-30

**Authors:** Veronika M. Cottontail, Elisabeth K. V. Kalko, Iain Cottontail, Nele Wellinghausen, Marco Tschapka, Susan L. Perkins, C. Miguel Pinto

**Affiliations:** 1 Institute of Experimental Ecology, University of Ulm, Ulm, Germany; 2 Institute of Medical Microbiology and Hygiene, University of Ulm, Ulm, Germany; 3 Smithsonian Tropical Research Institute, Balboa, Panama; 4 kbo-Isar-Amper-Klinikum, Taufkirchen (Vils), Germany; 5 Gaertner & Colleagues Laboratory, Ravensburg, Germany; 6 Sackler Institute for Comparative Genomics, American Museum of Natural History, New York, New York, United States of America; 7 Department of Mammalogy, American Museum of Natural History, New York, New York, United States of America; 8 The Graduate Center, City University of New York, New York, New York, United States of America; 9 Centro de Investigación en Enfermedades Infecciosas, Pontificia Universidad Católica del Ecuador, Quito, Ecuador; Tulane University, United States of America

## Abstract

The *Trypanosoma cruzi* clade is a group of parasites that comprises *T. cruzi* sensu lato and its closest relatives. Although several species have been confirmed phylogenetically to belong to this clade, it is uncertain how many more species can be expected to belong into this group. Here, we present the results of a survey of trypanosome parasites of the bat *Artibeus jamaicensis* from the Panamá Canal Zone, an important seed disperser. Using a genealogical species delimitation approach, the Poisson tree processes (PTP), we tentatively identified five species of trypanosomes – all belonging to the *T. cruzi* clade. A small monophyletic group of three putative *Trypanosoma* species places at the base of the clade phylogeny, providing evidence for at least five independent colonization events of these parasites into the New World. *Artibeus jamaicensis* presents a high diversity of these blood parasites and is the vertebrate with the highest number of putative trypanosome species reported from a single locality. Our results emphasize the need for continued efforts to survey mammalian trypanosomes.

## Introduction

How to separate one species from the other – species delimitation – is a central problem in organismal biology, and the complexity of the argument increases when dealing with potentially cryptic species. Usually, species delimitation relies on morphological characters and in similarity of DNA sequences, and often the chosen criteria can be arbitrary and misleading. This is particularly true for eukaryotic microbes where morphologies could be conserved and where distinctive characters are scarce or at least not easy to obtain [1]. Novel approaches that are based exclusively in gene genealogies, such as coalescent species delimitation may offer an alternative to this long-standing problem [2]. Recently, Poisson tree processes (PTP), a new model for coalescent species delimitation has been proposed [3]. Here we are interested in using PTP to explore the diversity of trypanosome parasites in a common Neotropical bat species, *Artibeus jamaicensis*.

Trypanosomes are protozoan blood parasites that use all classes of vertebrates as reservoir hosts, and certain blood-feeding invertebrates (e.g., cimicid bugs, leeches, triatomine bugs, tsetse flies) as vectors [4]. One of the best known trypanosome species is *Trypanosoma cruzi*, which causes Chagas disease. This species has several close relatives that together form a monophyletic group known as the *T. cruzi* clade [5], [6]. Despite recent progress [6]–[8], the evolutionary history, including the overall diversity and biogeographic patterns of this clade are far from understood. Several species have been confirmed phylogenetically to belong to this clade, but it is uncertain how many more can be expected within this group. Because of this uncertainty, biogeographic inferences regarding the origin of the clade and posterior dispersal events may be inaccurate.

Here we explore the diversity of trypanosome parasites in *Artibeus jamaicensis* from the Panama Canal Zone. Using PTP, a fast coalescent species delimitation approach, we recovered 5 different putative species of trypanosomes, all belonging to the *T. cruzi* clade [6], [8]. We discuss the impact of these findings on the biogeography and taxonomy of this important clade of parasites of mammals.

## Materials and Methods

### Ethics Statement

Permits for field research and exporting of samples were granted to VMC by the Panamanian Autoridad Nacional del Ambiente (# SEX/A-145-05). Manipulation of bats and procedures of Data collection followed a protocol of the Institutional Animal Care & Use Committee (IACUC) of the Smithsonian Tropical Research Institute (STRI).

### Collection of bat trypanosomes and genetic data

During field work conducted in 2005, we collected blood samples of 216 *Artibeus jamaicensis* in the Panamá Canal Zone, Panamá [9]. We extracted DNA, performed nested PCRs to screen for trypansomes and, for positive bats, we sequenced a fragment of the 18S ribosomal RNA gene [10]. We obtained a total of 81 sequences, trimmed to 543 bp after alignment. Detailed protocols, microscopy results, correlations with habitat fragmentation, and the finding of Tcbat have been published previously [9], [11].

### Sequences and alignment

We built two alignments, one for the 18S rRNA gene that included our generated sequences together with the data used in the most comprehensive study to date of the *T. cruzi* clade [8], and another only with published sequences of the gGAPDH gene [8]. We constructed alignments using MUSCLE [12] within Geneious v. 6.1.8 [13], and manually edited obvious misplacements. GenBank accession numbers and codes of the samples used are presented in Table S1.

### Phylogenetic analysis

We concatenated both gene alignments with SequenceMatrix v. 1.7.9 [14]. For this concatenated matrix we selected the best model and partition scheme using Partition Finder 1.1.1 [15]. We divided the data into four data blocks, one for the 18S rRNA gene, and three for the gGAPDH gene – one for each codon position. We chose the model following the Bayesian information criteria scores, which suggested grouping all blocks under a single partition with the model GTR+I+G We ran Bayesian and maximum likelihood analyses in MrBayes v. 3.1.2 [16] and RAxML v. 8.0.12 [17], respectively. We set the Bayesian analysis with two independent runs with 1 cold and 3 heated chains, for 10 million generations, sampling the chains every 100 generations. The analysis was allowed to run until reaching stationarity (stopval set at 0.01) and confirmed by MrBayes' potential scale reduction factor values close to 1.00. At the end of the run, 10% of the generated trees were discarded as burn in. For the maximum likelihood analysis, we used the option GTRCATI, which implements the CAT approximation – an optimization of the parameter Gamma – but the final tree was evaluated with the traditional GTR+I+G model. We estimated nodal support with posterior probabilities for the Bayesian analysis and with 1,000 bootstrap pseudo replicates for the maximum likelihood analysis.

### Species delimitation

We used the Poisson tree processes (PTP) model for species delimitation [3] to infer the most likely species numbers in our Panama data and the entire *T. cruzi* clade. PTP is an operational criterion of a gene coalescent view of the phylogenetic species concept [18]. The PTP method outperforms the commonly used GMYC model [19], possibly because PTP models the speciation rate directly from the number of substitutions in a non-ultrametric phylogeny, while GMYC uses time from an ultrametric tree, which is a computationally expensive and error-prone practice [3].

**Figure 1:**
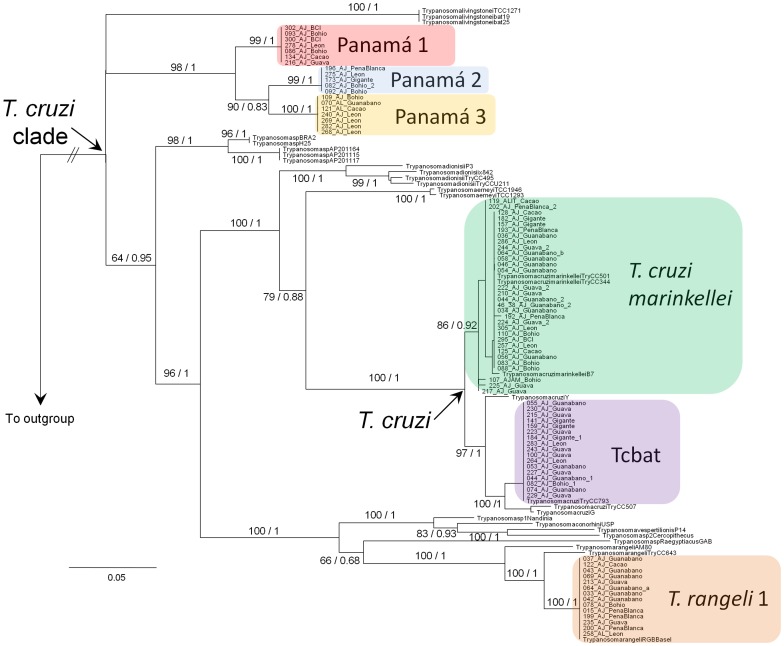
Phylogeny of the *Trypanosoma cruzi* clade. Maximum likelihood tree constructed with concatenated 18S rRNA and gGAPDH genes. Numbers on the branches represent support values corresponding to bootstrap replicates (right) and Bayesian posterior probabilities (left). Clades highlighted with colored boxes correspond to trypanosomes detected in Panamá. Collapsed branches at the base of the phylogeny indicate low support for the placement of the three Panamian lineages. The symbol//on the branch to the outgroup indicates a shortened branch. GenBank accession numbers of the samples used in this phylogeny are provided in Table S1.

We ran a PTP species delimitation analysis in the bPTP web server [3]. As input, we used a maximum likelihood phylogeny of the concatenated dataset, estimated as above but with a reduced number of terminals (n =  28), because PTP tends to overestimate the number of recognized species when there is uneven sampling of individuals per species [3]. In our matrix most of our terminals belonged to *T. cruzi* (Figure 1), so first, we removed all identical sequences and later we pruned additional members of *T. cruzi* that showed little variation in our dataset – mostly belonging to Tcbat and *T. cruzi marinkellei*. We ran the PTP analysis for 200,000 MCMC generations, with a thinning value of 100, a burn-in of 25%, and opted for removing the outgroup to improve species delimitation. We visually confirmed the convergence of the MCMC chain as recommended [3].

### Mapping of New World invasions

Following previously published geographic records [4], [6], [8], [20], we determined the number of invasions of members of the *T. cruzi* clade to the New World by identifying the branches containing species with representatives in the Americas.

## Results

### Phylogenetic relationships

The topology of both the Bayesian and maximum likelihood trees largely agree, except with the placement of the most basal species of the *T. cruzi* clade, which was either *Trypanosoma livingstonei*, the monophyletic group consisting of three lineages of Panamanian bat parasites, or the group formed by the two Australian trypanosomes (Figure 1). This is reflected in the low bootstrap support values and Bayesian posterior probabilities at the base of the tree, while the support values at other branches of the tree are high for most part.

### Species delimitation

**Figure 2:**
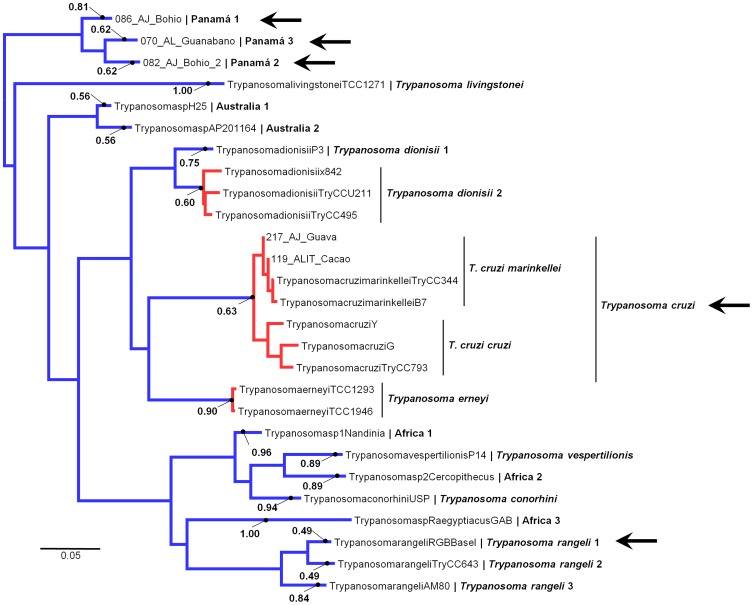
Putative species delimitation of members of the *Trypanosoma cruzi* clade. Maximum likelihood phylogeny with Bayesian support values presented for all 18 lineages recognized as species for the PTP analyses. Monophyletic groups in red indicate a single putative species as well as terminal branches in blue. Names of terminals indicate codes of the samples. Names in bold after a | symbol are taxonomic or geographic identifiers of the putative species. Arrows indicate the 5 putative species found in *Artibeus jamaicensis* in Panamá.

The PTP model identified a total of five putative species of *Trypanosoma* in the samples of *Artibeus jamaicensis* from Panamá (Figure 2). Three of these putative species clustered together forming a monophyletic group positioned in the periphery of the *T. cruzi* clade and the other two were identified as *T. cruzi* and *Trypanosoma rangeli*. Including the putative species detected in Panamá, the PTP model detected 18 species as members of the *T. cruzi* clade. Both, *T. cruzi cruzi* and *T. cruzi marinkellei* were recognized as a single species and the PTP model also recognized at least three putative species within what is known as *T. rangeli* and two putative species within *Trypanosoma dionisii* (Figure 2).

### *T. cruzi* clade invasions to the New World

**Figure 3:**
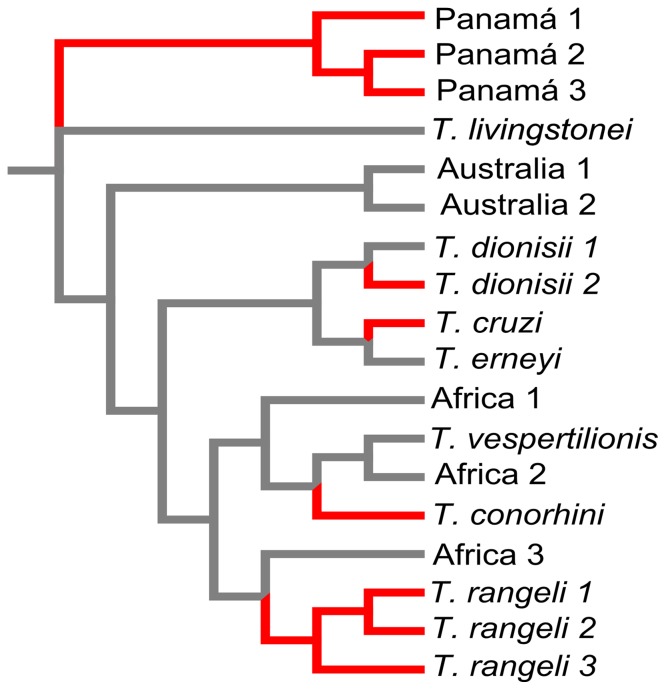
Five independent colonization events of *Trypanosoma cruzi* clade members to the New World. Cladogram of species belonging to the *Trypanosoma cruzi* clade, depicting in red are the branches that indicate dispersal events to the Americas with grey branches denoting the species that are not present in the New World. Note that *T. dionisii* 2 and *T. conorhini* are distributed in the New and Old Worlds [20], [43]. Branch lengths in the cladogram are not proportional to divergence; for branch length estimates refer to Figures 1 and 2.

We recognize a total of five independent invasions to the New World (Figure 3). Two invasions correspond to species with multiple-continent distributions: *T. dionisii*, and *T. conorhini.* Two other invasions produced small radiations of three putative species each: the basal group of Panamian samples and the group of putative species assigned to *T. rangeli*. Another invasion gave rise to *T. cruzi* and all of its subspecific lineages.

## Discussion

Our finding of five putative trypanosome species in a single bat host species from a small geographic area is remarkable for the implications that it has for understanding the diversity, evolution, and biogeographic origins of mammalian *Trypanosoma* in the New World.

### Diversity

*Artibeus jamaicensis* is a common bat species in the Neotropical forests, and it is considered a key species for its ecological services as a seed disperser [21]. Here we demonstrate that *A. jamaicensis* carries the largest number of putative trypanosome species reported for a vertebrate host in a single locality; no other vertebrate species has been found to carry a higher number of trypanosome species at one particular geographic area [8], [22]–[25]. Potentially, this high diversity of parasites in this host species is driven by several factors. *Artibeus jamaicensis* is the most common bat species in the area [26], and Barro Colorado has a high diversity of triatomine insect vectors (e.g., *Panstrongylus geniculatus*, *Panstrongylus rufotuberculatus*, *Rhodnius pallescens*, *Microtriatoma trinidadensis*), which have been found in association with bats at other localities [27]. In addition, the location of Barro Colorado relatively close to the equator may suggest that trypanosome parasites – at least of bats – could follow the general latitudinal gradient of diversity, where higher numbers of species are recorded near the equator and the numbers decrease towards the poles [28]. Both bats and their bat flies also show this latitudinal diversity gradient [29], [30].

Other than the biotic drivers reported above, undoubtedly the detection and analytical methods that we employed also played a role in finding a high number of putative trypanosome species. First, detection of trypanosomes by PCR is more sensitive than detection by microscopy alone [31]. Also, DNA sequencing and phylogenetic analyses with comparative sequences allow more reliable identification of species than morphological characters or the “one-host-one-species paradigm” [32]. Moreover, species delimitation models are efficient and theoretically sound, and become a good alternative given the intrinsic subjectivity in delimiting species using genetic distances or arbitrary morphological groupings [33].

### Taxonomy

Our results indicate that the *T. cruzi* clade has more species than previously reported, especially in the New World, and this high diversity warrants further surveys and taxonomic study. More genetic sampling of taxa will help to resolve new and outstanding taxonomic issues within this clade. It seems that the most pervasive problems are the high number of species names available [22] and the lack of genetic data corresponding to several of these names. For instance, the Panamanian putative species 1, 2, and 3 form a monophyletic clade, which indicates that their morphologies may be very similar. It's possible that some of these lineages could be synonymous with *Trypanosoma leonidasdeanei* or *Trypanosoma pessoai* [38] from Costa Rican bats. Moreover, it is possible that the strain called Z or “*T. c. marinkellei* III?” [39] – a trypanosome highly divergent from *T. cruzi marinkellei* in electrophoretic patterns and nucleotide sequences [39], [40] – could belong to one of our Panamanian putative species. Unfortunately, there are not publically available DNA sequences of this lineage.

A particularly interesting taxonomic issue is the case of *T. rangeli*, a parasite also found in humans, which we show is actually composed from at least 3 different putative species (Figure 2). In the last two decades, the taxonomy of *T. rangeli* has been reviewed using molecular methods and, because shallow divergences were reported, *Trypanosoma leeuwenhoeki*, *Trypanosoma legeri*, *Trypanosoma minasense*, *Trypanosoma preguici*, and *Trypanosoma saimiri* were all recommend to be treated as junior synonyms of *T. rangeli* [41], [42]. The shallow divergences reported within *T. rangeli* in these previous studies probably were the result of the removal of all columns of the alignment that contain gaps, which, paradoxically, are the more informative columns of the 18S rRNA gene alignments, thus resulting in the observed reduced variation. Current software such as RAxML [17] treats the gaps as undetermined characters “Ns”, and the entire alignment column is kept.

### Biogeography

With this new phylogeny we recognize five invasions of *T. cruzi* clade members to the New World (Figure 3). This number is greater than has been detected in previous phylogenetic analyses [6]–[8], and it suggests that we would find signs for an even higher number of invasions if more thorough bat trypanosome surveys are conducted. Also, it is interesting that most of these invasions did not generate more diversity. The only exceptions are the sub-clade containing *T. rangeli* and the three putative species from Panama that seem that have undergone small radiations (Figure 2).

Two invasions appear to be rather recent and while one is the result of natural dispersal, the other seems facilitated by human activity. *Trypanosoma dionisii* is a parasite of bats that has been recorded in Europe and South America, and it has been hypothesized that the colonization of South America has occurred relatively recently, probably by vagrant bats, since there are no species of bats shared between the Americas and Europe [20]. In addition, *T. conorhini* uses Old World rats and vectors as hosts – *Rattus* spp. and *Triatoma ribrofasciata*, respectively – and its presence in the New World could be explained by the recent human-mediated introduction of rats and associated parasites [43]. This is highly plausible since *Trypanosoma lewisi*, also a parasite associated with rodents of the genus *Rattus*, has been reported in several continents including oceanic islands [36], [44], [45].

In our phylogeny, it is not possible to determine the branching order at the base of the *T. cruzi* clade due to the low support values (Figure 1); however, either the group formed by Panama 1, 2 and 3 or *T. livingstonei* should be at the base of the tree. Because the most basal species of the *T. cruzi* clade are distributed on different southern continents, including Africa, there is support for a wider Gondwanan origin of the clade, rather than a more limited South American-Australian origin [5]. Further, trypanosome surveys may help discovering more basal trypanosome lineages that might allow a finer inference of the ancestral distribution area of this clade by limiting the ancestral origin to a single southern continent. Nonetheless, all of the potential basal species of the *T. cruzi* clade are parasites of bats; which further supports a likely origin of this clade in bats, as well as the bat-seeding hypothesis that indicates that bats are the main hosts of the *T. cruzi* clade. Episodic host switches towards terrestrial mammals may have occurred along the evolution of this clade including the host switches that ultimately gave rise to the pathogen *T. cruzi* that causes Chagas disease in humans [43].

### Outlook into future trypanosome surveys

We advocate the use of DNA sequence data and coalescent-based methods for species delimitation because these will speed up biological discovery, especially of microscopic organisms with plastic morphologies. We recommend that species descriptions of new taxa continue to include morphological analyses, since at least major groupings can be identified reliably through gross morphological characters and measurements [34] and this may allow the tracking of names available from older literature. In this study, the recognition of *T. cruzi* as a single species despite the pronounced divergence between *T. cruzi cruzi* and *T. cruzi marinkellei*, may indicate that the PTP model, applied on trees of the 18S rRNA and gGAPDH genes, is a conservative approach that is not oversplitting taxa. Species delimitation results may be highly dependent of the loci used; a fast evolving gene might recognize a higher number of putative species than a slow evolving marker. Also, it is important to emphasize that computational species delimitation methods have different assumptions and may target different areas of the parameter space that is relevant for delimiting species (e.g., divergence time, shifts in diversification), thus resulting in different outputs depending on the methods used [33]. The genes used in this study are slow evolving, and PTP model has shown to be reliable at species delimitation [3]; thus, we may have confidence in this approach as an efficient way to rapidly delimit putative species, at least preliminarily, until more genetic data (e.g., dozens of loci) could be gathered and several more robust delimitation methods could be applied (e.g., Bayesian species delimitation, bpp) [2], [33].

We also suggest more efforts to survey trypanosomes of mammals, either by conducting field expeditions targeted on collecting trypanosome material [9], [35], or by surveys of trypanosome DNA in mammalian specimens and tissues deposited at museum collections [36], [37]. Sustained efforts should be devoted for trypanosome sampling in Africa and the Americas to truly understand the diversity of this clade. In the Americas in particular, it would be important to survey localities in Central and North America, and in South America on the western side of the Andes and the Guiana Shield.

## Supporting Information

Table S1 **GenBank accession numbers of the samples used.** Codes in bold represent the new sequences generated for this study, hyphens indicate absence of the gGAPDH sequences for the respective samples.
(XLSX)
